# Puberty in female wild boar (*Sus scrofa*) in Sweden

**DOI:** 10.1186/s13028-016-0236-1

**Published:** 2016-09-27

**Authors:** Anna Malmsten, Anne-Marie Dalin

**Affiliations:** Division of Reproduction, Department of Clinical Sciences, Swedish University of Agricultural Sciences, Box 7054, SE-750 07 Uppsala, Sweden

**Keywords:** Ovaries, Uterus, Follicles, Corpora lutea, Wildlife management, Reproduction

## Abstract

**Background:**

Since the re-appearance of wild boars in Sweden in the 1970s, the population has increased. Besides having large litter sizes, puberty at an early age is considered as an important factor contributing to the high reproductive potential of wild boar. Although controversial, supplemental feeding is applied to varying extent throughout the wild boar range in Sweden, and its effect on wild boar reproduction is debated. The aim of this study was to investigate the proportion of post-pubertal female wild boar gilts in a population subjected to supplemental feeding, in relation to age, weight, and season. Also, the effect of another definition of puberty (based upon follicular size) on the outcome of the proportion of female wild boar gilts considered to be able to reproduce in a population was illustrated. Between Jan 2013 and Dec 2015 reproductive organs from 592 female wild boars, were collected. Ovaries and uterus were macroscopically examined, and reproductive stage was determined by the presence of ovarian structures and uterus characteristics. Age was estimated using tooth eruption and tooth replacement, and weight was noted. A subset of 175 female wild boars, aged 5–15 months, was included in this study. An animal was considered to be post-pubertal if the ovaries contained one or more *corpora lutea* (CL) or if the uterus showed signs of previous pregnancy (presence of enlarged blood vessels in the cut surface between the mesometric ligament and the uterus).

**Results:**

In total, 29 (16.6 %) animals were classified as post-pubertal. Field dressed weight ranged from 20.6 to 65.3 kg. Season, weight, and age class significantly influenced the variation in proportion of post-pubertal females. Post-pubertal animals were found in autumn, winter, and spring, but not in the summer season. Another definition of puberty, based on follicle size, lead to different result on proportions of animals considered to have passed puberty.

**Conclusions:**

Season, weight, and age significantly influenced the variation in proportion of post-pubertal wild boar females. The proportion of post-pubertal animals increased with age and weight. However, weight is probably a better proxy for puberty than age group is. The proportion of post-pubertal females also increased from summer to spring suggesting a seasonal reproductive pattern. Different definitions of puberty will result in various outcomes, which high-lights the importance of using adequate definition of puberty.

## Background

From the 1980s to the 2010s populations of wild boar (*Sus scrofa*) have increased remarkably in Europe [1, 2], and this increase has had widespread ecologic and economic consequences. In forested areas, the increase can affect plant diversity and vegetation composition [3]. In cultivated areas, crop damage of varying severity is frequent [4, 5]. Moreover, the wild boar is also considered a reservoir of various pathogens (e.g. *Mycobacterium bovis* and Hepatitis E virus) that may infect other wildlife, domestic animals, and humans [6, 7].

In addition to the behavior of wild boar (flexible nutrition, and habitat selection etc.), the main reason for the rapid population increase is thought to be due to high reproductive potential [8]. Besides having large litter sizes, puberty at an early age is another important factor contributing to the high reproductive potential. In domestic pigs, lifetime reproductive success and longevity is dependent on several factors such as age at puberty and first mating [9, 10] as well as subsequent factors affecting body condition [11]. The sooner in life reproduction starts, the higher the lifetime offspring production may be. However, there are also disadvantages related to the start of reproduction at an early age because the female may be less physically robust.

In Sweden, since the wild boar reappeared in the 1970s when few individuals escaped enclosures, the population has increased. In 2012 and 2013, 84,000–97,000 animals were culled, per year during hunting [12]. Although controversial, supplemental feeding is applied to a varying extent throughout the wild boar range in Sweden, and its effect on wild boar reproduction is debated. Hunters and farmers claim that supplemental feeding make wild boar gilts pubertal at an earlier age than what is considered to be natural. Other statements include the ability of young gilts to reach puberty throughout the year, which also is considered to be atypical for a short day breeder such as the wild boar [13].

In domestic female animals the definition used for an animal to be able to reproduce, i.e. having attained puberty, is that it should show external signs of oestrous and ovulate [14]. Once puberty has been reached the female should be able to show regular oestrous cycles if not pregnant. In Swedish domestic crossbreed pigs, gilts usually reach puberty at 6–7 months of age [15, 16]. Season can influence reproduction of domestic pigs [17–20], e.g. puberty [21] and age at first mating [22].

In free-ranging wild boar it is difficult to monitor if a gilt shows oestrus and exhibits regular oestrus cycles. Therefore, the majority of studies of puberty in free-ranging female wild boar gilts are based on post-mortem macroscopic examinations of the reproductive organs. Yet, the definition of puberty in these studies varies, which make comparisons difficult. Most commonly signs of ovulation, which is presence of one or more *corpora lutea* (CL), is used to define if the female wild boar has reached puberty [4, 23]. However, others declare that ‘puberty is assumed in individuals with follicles more than 3 mm in diameter [24], i.e. without the necessity of the presence of one or more CL. In addition, wild boar females below 1 year of age have been categorized as being cyclic, i.e. having passed puberty, if found with follicles of 6 mm in diameter or larger [25]. The exact timing of when a female passes puberty cannot be detected by macroscopic examination of reproductive organs. This method can only result in proportions of animals in a population that have passed puberty. If the exact timing of puberty needs to be studied, other methods such as repeated fecal [26] or blood [27, 28] sampling for progesterone analyses of fenced animals can be used. These methods are harder, or impossible to apply in free-ranging animals.

The aim of this study was to investigate the proportion of post-pubertal female wild boar gilts in a population subjected to supplemental feeding, in relation to age, weight, and season based on ovarian structures (presence of CL) and uterus characteristics. Also, the effect of another definition of puberty (based upon follicular size) on the outcome of the proportion of female wild boar gilts considered to be able to reproduce in a population were investigated.

## Methods

As part of a larger study of female wild boar reproduction, data were collected from hunter-harvested wild boars in seven hunting estates in the southern and middle part of Sweden. Information of habitat composition, feeding practices, estimated population sizes (wild boar and other ungulates) was collected from each estate. The area of the estates varied between 10 and 87 km^2^, the density of wild boars were estimated at 5–40 per km^2^, and the amount of supplemental feeding was 125,000–300,000 kg/year distributed throughout the year. All estate data was sampled between January 2013 and December 2015, during which culled female wild boar samples were collected. Hunting methods used were drive hunts and stalking. In drive hunts, the hunters are posted at stands around a tracked area. The wild boars are driven towards the hunters by beaters with dogs. This method was used during the main hunting season between October and February and often resulted in a big hunting bag (30–100 animals per day). Stalking was used during spring and summer and the animals were most often shot while feeding on an open field.

In Sweden, wild boar hunting is allowed 24 h and hunting with dogs is allowed between 1 of August and 31 January. The hunting season of adult wild boars is between 16 April and 15 February. Hunting of yearlings is allowed the year around but females with dependent piglets are protected. The sampling effort was for practical reasons biased towards the main hunting season (October–February).

Whole body weight (BW) and field dressed weight (FDW, weight of the eviscerated animal with skin) were noted. In animals where only BW was noted, FDW was estimated using the relationship FDW = −1.855 + 0.810*BW [Pers. comm. N. Lundeheim]. Date of harvest was noted and the year was divided into seasons; spring (March–May), summer (June–August), autumn (September–November), and winter (December–February).

Age was estimated using tooth eruption and tooth replacement [29]. In this study, animals between the ages of 5 and 15 months were included. The age span used was based on previous studies of puberty in domestic pig [16]. Animals where divided into three age classes according to Matschke [29]; 5–8 months, 7.5–12 months, and 12–15 months. The uterus and ovaries were macroscopically examined. The uterus was cut open and the color and thickness of the endometrium was examined. Signs of pregnancy (embryos/fetuses) or signs of previous pregnancy were noted. Presence of enlarged blood vessels in the cut surface between the mesometric ligament (*ligamentum mesometrium*) and the uterus indicated previous pregnancy [Pers. comm. E. Persson; 30]. Luteal structures (*corpora hemorrhagica*, active CL, and regressing CL) were cut open, counted and the diameter was measured to the nearest millimeter. The number and diameter of luteal structures, and follicles (≥4 mm) found in the ovaries was noted. An animal was considered to be post-pubertal if the ovaries contained one or more CL [4, 23], or if the uterus showed signs of previous pregnancy [30]. In order to illustrate how another definition of puberty may influence the outcome of the study, we compared the proportions of post-pubertal animals (according to the definition above) with the proportion of animals found with CL or follicles ≥4 mm [24].

### Statistical analyses

Because the response variable is binary, data were analyzed using as generalized linear models (see e.g. [31]) with a binomial distribution and a logistic link function. The Glimmix procedure of the SAS (2009) package was used [32]. The relation between probability of puberty and age, weight and season was of primary interest. Weight was available as a continuous variable, while age was assessed roughly into three classes based on dental status.

In the model building process, models were compared based on the Alailke Information Criterion, AIC [33]. Preliminary analyses suggested that weight, but not age class, should be used as a continuous covariate. In a first model, all interactions were included, but they could all be removed from the model, in a stepwise fashion, based on the AIC. Thus, the final model can be written as

$$ {\text{logit}}( {{\text{p}}_{\text{ijk}} }) = \mu + \alpha_{\text{i}} + \gamma_{\text{j}} + \beta {\text{x}}_{\text{k}} $$

where α_i_ denotes season *i*, γ_j_ denotes age class *j*, and x_k_ is the weight of boar *k*.

The assumptions used were checked by preparing appropriate residual plots; no apparent deviations from the assumptions could be detected. Pairwise comparisons were adjusted for multiplicity using Tukey’s method.

Fisher’s exact test in R [34] was used to compare the proportion of post-pubertal female wild boars and the proportion of animals found with CL or follicles ≥4 mm, in different age classes.

## Results

During the sampling period, 592 female wild boars of different ages were culled and sampled. Of these, 175 individuals met the requirements to be included in this study, which were complete sets of reproductive organs, age span 5–15 months, and weight information. Field dressed weight ranged from 20.6 to 65.3 kg.

**Fig. 1:**
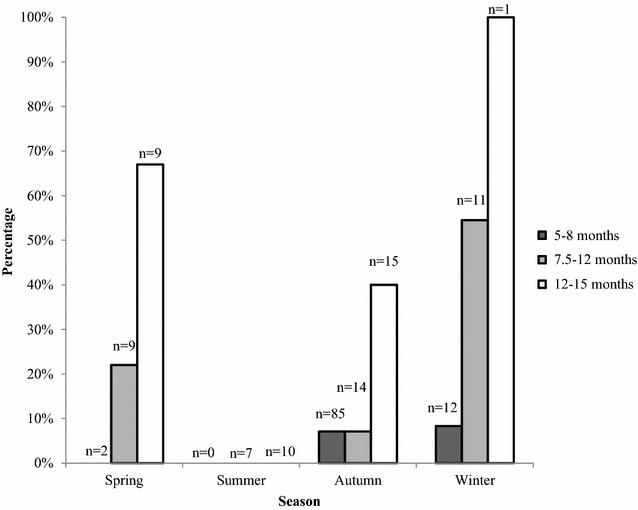
The proportion of post-pubertal female wild boars according to age class and culling season

In total, 29 (16.6 %) of the examined animals were considered as post-pubertal, and one or more CL were found in all but one of these animals. Of these, 15 were pregnant, nine were cyclic, and four had regressed CL and uterus with signs of previous pregnancy. One animal had no CL structure but the uterus showed signs of previous pregnancy. The mean number of CL in cyclic animals (n = 9) was 4.7 (range 2–8) and in pregnant animals (n = 15) 5.6 (range 2–9). The mean number of embryos/fetuses was 4.8 (range 2–9) in 13 pregnant animals in which pregnancy had proceeded more than 3 weeks. Counting the exact number of embryos before 3 week of pregnancy (n = 2) is difficult at only macroscopic examination. Post-pubertal females were found all seasons but the summer (Fig. 1).

**Fig. 2:**
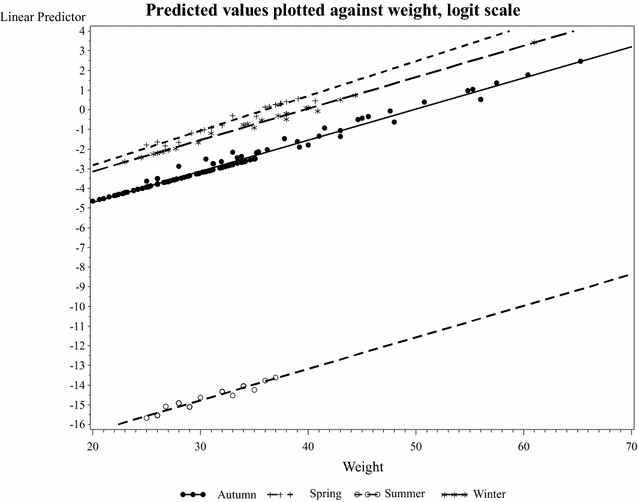
Predicted values of puberty in female wild boars, plotted against body weight and divided by season

In a first statistical analysis, age class, weight and season were analyzed separately. All of them showed significant relations to puberty: weight (P < 0.0001); season (P = 0.0079) and age class (P = 0.0006). In the joint model, season (P = 0.0395) and weight (P = 0.0002) were significant, but not age class (P = 0.7398). This is because age and weight are highly correlated, and weight is probably a better proxy for puberty than age class is. In Fig. 2, the fitted values from the model are plotted against body weight, with one curve for each season.

There were significant differences of proportions of post-pubertal animals and animals found with CL or follicles ≥4 mm, in different age classes (P < 0.001). The different proportions are presented in Table 1.

**Table 1:** Numbers and proportion of Swedish female wild boars in puberty in different age classes, according to two previously used definitions

Age class (months)	N	Females attained puberty (%) according to definition A^a^	Females attained puberty (%) according to definition B^b^
5–8	99	7 (7.1)	79 (79.8)
7.5–12	41	9 (22.0)	37 (90.2)
12–15	35	13 (37.1)	34 (97.1)
Total	175	29 (16.6)	150 (85.7)

^a^Presence of corpus luteum or previous signs of pregnancy in uterus [4, 23]

^b^Presence of corpora luteum or ovarian follicles ≥4 mm [24]

## Discussion

As expected, this study shows that the weight of the female wild boar and as well as the season influenced the variation in proportion of post-pubertal females. The body composition (fat content) has been shown to affect age at puberty in various species [30, 35, 36], including wild boar [25]. To start reproducing, females of most mammal species need to pass a body mass and composition threshold. Females that pass this threshold early, due to favorable conditions such as high feed availability, will thus start reproducing at a young age.

In this study, we found higher proportions of gilts that had passed puberty in the winter and spring months than during the summer and the autumn. No post-pubertal animal was found in the summer season. However, females, regardless of age, accompanied by piglets are prohibited to hunt which might have affected the result. Moreover, the examined animals were culled during hunting and the sampling effort was biased towards the main hunting season October–February which might have influenced the results. Still, the wild boar is a “short day breeder” [13], and we believe that the result mostly are explained by the stimulatory effect of the short days of autumn and winter.

In wild boar, as in most other game species, population growth is dependent on the proportion of females that are able to reproduce. In a species where reproductive capability is reached at a young age, the population growth will be fast, especially in pluriparous species. Because of hunting constraints (not culling females accompanied by piglets), animals included in this study cannot be considered as a random sample. Thus, an underestimation of the proportion post-pubertal animals is possible. Only 11.4 % of the animals aged <12 months were classified as post-pubertal. In southern and middle part of Sweden, the feed availability (excluding supplemental feeding) is high for wild boar, both as crops and natural feed. During periods of natural feed shortage, as in winter, supplemental feeding is extensive. Considering the food availability, one would suspect that the proportion of post-pubertal animals would have been higher and more in accordance to other studies [4, 23] of wild boars using the same definition of puberty (occurrence of ovarian CL) as in present study. Extensive supplemental feeding has earlier been discussed as one of the main causes of passing puberty at a young age [23]. Cellina [23] reported that in Luxemburg, 24 % of supplementary fed wild boar females aged <12 months were post-pubertal, and the youngest was four months old. No females younger than 5 months of age were investigated in the present study, but based on the low proportion (7.1 %) of post-pubertal gilts aged 5–8 months it is unlikely that younger females had passed puberty to a significant extent. However, due to the wide age category (months), it is impossible to know whether pregnant animals in this study had reached puberty within the noted age category or at a younger age.

Ahmad et al. [4] showed a very high proportion (25/32) of gilts aged 4–7 months in Pakistan with CL, which differ from wild boars in this study as well as from a study in Luxemburg [23]. Thus, wild boars in Pakistan seem to be more similar to domestic pigs than Swedish wild boar. Therefore, the genetic background of wild boars may be of importance for the pubertal age as seen in the domestic pigs [32] and need to be studied in different regions/countries. Domestic, crossbred pigs normally reach puberty at 6–7 months of age [15, 16] and if gilts have not reached puberty at the age of 8 months they are considered to have delayed puberty [16]. This illustrates one substantial difference between domestic European pig breeds and wild boar. As presented in Table 1, different definitions of puberty have a major effect on the result of the proportion of female wild boar gilts considered to have passed puberty in a population as well as in different age classes. In the present study, 79.8 % of the examined wild boars aged 5–8 months was found with follicles ≥4 mm only or with CL and would have reached puberty according to this definition. This result is similar to the one of Gethöffer et al. [24] who found an 80 % chance of juveniles having attained sexual maturity at 8 months of age using that definition. Gethöffer et al. [24] based their definition of puberty on studies on the viability of the oocyte in various follicle sizes. A great caution must be taken when using such definition of puberty because a large number of follicles will become atretic before the animal has reached puberty, with no regards to whether the oocyte is viable or not at a certain stage. When using only follicle size, the proportion of wild boar females considered being capable to reproduce will be overestimated.

Presence of CL shows that ovulation indeed has occurred. This is the definitions of puberty used for domestic pigs [16, 37] and other domestic species when examining reproductive organs. It is also important to include uterus characteristics when evaluating if an animal has reached puberty or not. In the present study one of the animals that had passed puberty had no CL (being anoestral) and would have been ‘missjugded’ as non-pubertal using definitions that only consider ovarian findings. In addition, by examining the uterus it is possible to distinguish gilts in anoestrous from sows in anoestrus (passed pregnancy), as well as pregnant females (>2 weeks of pregnancy) from females that are cyclic.

## Conclusions

Season, weight and age significantly influenced the variation in proportion of post-pubertal females. The proportion of post-pubertal animals increased with age and weight. However, weight is probably a better proxy for puberty than age group is. The proportion of post-pubertal females also increased from summer to spring suggesting a seasonal reproductive pattern. Different definitions of puberty will result in various outcomes, which high-lights the importance of using adequate definition of puberty.
